# Characterization of *BiP* Genes from Pepper (*Capsicum annuum* L.) and the Role of *CaBiP1* in Response to Endoplasmic Reticulum and Multiple Abiotic Stresses

**DOI:** 10.3389/fpls.2017.01122

**Published:** 2017-06-28

**Authors:** Hu Wang, Huanhuan Niu, Yufei Zhai, Minghui Lu

**Affiliations:** College of Horticulture, Northwest A&F UniversityShaanxi, China

**Keywords:** *BiP*, pepper, *Arabidopsis*, ER stress, abiotic stresses, UPR pathway

## Abstract

Adverse environmental conditions have a detrimental impact on crop growth and development, and cause protein denaturation or misfolding. The binding protein (BiP) plays an important protective role by alleviating endoplasmic reticulum (ER) stress induced by misfolded proteins. In this study, we characterized three *BiP* genes (*CaBiP1, CaBiP2*, *and CaBiP3*) in pepper, an economically important vegetable and spice species. The role of *CaBiP1* in plant tolerance to ER stress and adverse environmental conditions (including heat, salinity, osmotic and drought stress) were investigated. All the expected functional and signaling domains were detected in three BiP proteins, but the motifs and exon-intron distribution differed slightly in CaBiP3. *CaBiP1* and *CaBiP2* were constitutively expressed in all the tested tissues under both normal and stressed conditions, whereas *CaBiP3* was mainly expressed following stress. Silencing of *CaBiP1* reduced pepper tolerance to ER stress and various environment stresses, and was accompanied by increased H_2_O_2_ accumulation, MDA content, relative electric leakage (REL), water loss rate, and a reduction in soluble protein content and relative water content (RWC) in the leaves. Conversely, overexpression of *CaBiP1* in *Arabidopsis* enhanced tolerance to ER stress and multiple environment stresses, as demonstrated by an increase in germination rate, root length, survival rate, RWC, the unfolded protein response (UPR) pathway, and a decrease in water loss rate. Our results suggest that CaBiP1 may contribute to plant tolerance to abiotic stresses by reducing ROS accumulation, increasing the water-retention ability, and stimulating UPR pathways and expression of stress-related genes.

## Introduction

With advancing global warming, extreme weather events, especially high temperatures and droughts in arid and semiarid areas, pose an increasing threat to crop productivity. To develop crop varieties that better tolerate these adverse conditions, a greater understanding of the mechanisms involved in environment stresses is crucial (Thiry et al., 2016). In general, the growth and development of plants requires proteins to function in a normal manner, but the elaborate folding of proteins is easily disturbed by adverse environmental conditions, whereas it is well known that the accumulation of misfolded proteins is harmful to plant health (Howell, 2013).

The ER is the site of the protein secretory pathway in plant cells, and it is responsible for the folding and assembly of about one third of all cellular proteins (Deng et al., 2013). When misfolded proteins accumulate in the ER, the balance between folding pressure and folding capacity is broken, resulting ER stress (Wan and Jiang, 2016). During evolution, plants have developed a comprehensive mechanism to mitigate ER stress induced by adverse environmental conditions. One important strategy is up-regulating the transcription of ER chaperones to enhance the capacity of the protein folding machinery (Fanata et al., 2013).

Binding protein is a member of the Hsp70 family that include an HDEL or KDEL ER retention factor motif at the *C*-terminus (Denecke et al., 1991). BiP is one of the most abundant chaperones in ER lumen, and it has an ATP-binding domain and a protein- binding domain at the *N*- and *C*-terminus, respectively, the latter of which allows it bind to the hydrophobic surfaces of nascent proteins to protect them from aggregation via an ATP-dependent mechanism (Howell, 2013).

Under normal growth conditions, *BiP* genes are highly expressed in plant tissues and during plant developmental events with high cellular secretory activity and/or a high proportions of rapidly dividing cells, such as male and female gametogenesis (Maruyama et al., 2014, 2015). When plants are subjected to the ER stress induced by adverse environmental factors or ER stressors, such as heat in *Arabidopsis* (Deng et al., 2011), rice (Jung et al., 2013), and soybean (Zhang et al., 2015), drought in wheat (Zhu et al., 2014), tunicamycin (TM) exposure in *Arabidopsis* (Noh et al., 2003), dithiothreitol (DTT) exposure in rice (Wakasa et al., 2012), *BiP* genes are up-regulated via the UPR (Wan and Jiang, 2016). Once misfolded proteins accumulate in the ER, the bZIP28 and IRE1 branches of UPR are activated, and the transcription factors of bZIP28 and bZIP60 are released and progressively relocate to the nucleus to up-regulate UPR-related genes including *BiP* (Howell, 2013). Therefore, the up-regulation of *BiP* gene is a marker of the UPR (Cheng et al., 2015).

Overexpression of *BiP* genes also enhances plant tolerance to environment stresses. Yang et al. (2016) found that overexpression of *BiP* genes and the exogenous chemical chaperones sodium 4-phenylbutyrate (PBA) alleviated the ER stress induced by DTT and high temperatures. Overexpression of soybean *BiP* in tobacco conferred tolerance to water deficit during plant growth by preventing endogenous oxidative stress (Alvim et al., 2001), and similar results were observed in soybean (Valente et al., 2009). Leborgne-Castel et al. (1999) also found that overexpressing *BiP* in transgenic plants mitigated ER stress and reduced the UPR. In addition, silencing of *BiP* genes in tomato compromised Ve1-mediated resistance to *Verticillium dahlia* (Liebrand et al., 2014), and plants overexpressing *BiP* line displayed hypersensitivity to *Pseudomonas syringae* pv *tomato* in soybean and tobacco (Carvalho et al., 2014b).

Unlike mammals and yeast that possess only one copy in their genomes, plant genomes contain multiple highly conserved *BiP* genes, with six in rice (Sarkar et al., 2013) and tobacco (Denecke et al., 1991), three in *Arabidopsis* (Noh et al., 2003) and wheat (Zhu et al., 2014), two in soybean (Zhang et al., 2015), and 22 *BiP-like* genes are present in maize (Li et al., 2012). However, different *BiP* members exhibit different expression patterns. For instance, while *AtBiP-1* and *AtBiP-2* in *Arabidopsis* show significant basal expression in unstressed cells, *AtBiP3* is normally expressed at much lower levels but is highly induced under stress conditions (Noh et al., 2003). *OsBiP1* is constitutively expressed in various tissues in rice, whereas *OsBiP4* and *OsBiP5* appear not to be expressed in any tissue under normal conditions, but they are highly up-regulated following exposure to DTT (Wakasa et al., 2012). Therefore, characterizing the expression patterns and functions of *BiP* genes in response to abiotic stresses will greatly contribute to our understanding of plant tolerance to adverse environmental conditions.

In our previous study, we identified three *BiP* genes, *CaBiP1* (*CaHsp70-8*, CA01g00570), *CaBiP2* (*CaHsp70-7*, CA03g20120), and *CaBiP3* (*CaHsp70-10*, Capana08g001522), in the genome of pepper (*Capsicum annuum* L.), an economically important vegetable and spice crop (Guo et al., 2016). In the present study, we further analyzed the structure and expression pattern of these genes under normal and stressed conditions. Virus-induced gene silencing (VIGS) and overexpression analyses were performed to investigate the functions of *CaBiP1* in response to multiple abiotic stresses (including heat, salinity, osmotic, and drought stress) in both pepper and *Arabidopsis*. Our results provide insight into the function of BiP in the plant response to environment stresses.

## Materials and Methods

### Plant Materials and Growth Conditions

The R9 thermotolerant pepper line (introduced from the World-Asia Vegetable Research and Development Center, PP0042-51) and the *Arabidopsis* ecotype Col-0 variety were used in this study. Pepper seedlings were grown under normal conditions (26°C/20°C day/night, 200 μmol⋅m^-2^⋅s^-1^ illumination intensity, thermo- and photoperiod of 16 h light /8 h dark cycle, and 70 % relative humidity) in a controlled climate chamber, and *Arabidopsis* seedlings were grown under 22°C/18°C day/night conditions.

### Sequence Analysis of CaBiP Proteins

The amino acid sequences of CaBiP1, 2, and 3 were downloaded from the PGD^1^ (Qin et al., 2014) and PGP^2^ (Kim et al., 2014) pepper genome databases. *Arabidopsis* AtBiP amino acid sequences were obtained from Genbank^3^ (Noh et al., 2003) and rice OsBiP sequences were downloaded from Rice Genome Annotation Project^4^ (RGAP) (Sarkar et al., 2013).

Alignment of full-length BiP amino acid sequences from pepper, rice and *Arabidopsis* was performed using the online program of Clustal Omega^5^, and the phylogenetic tree was constructed using MEGA 6 with the neighbor-joining method, *p*-distance substitution model and 1000 bootstrap replicates (Tamura et al., 2013). Functional and signaling domains in CaBiPs were identified based on published *Arabidopsis* and rice literatures (Noh et al., 2003; Sarkar et al., 2013). The identification of conserved motifs in CaBiP proteins was carried out using the MEME program^6^ with the following parameters: normal motif discovery mode, maximum number of motifs = 9, a motif site distribution in which each gene has none or only one motif, and a motif width between four and 200 amino acids. Structural diagrams for exon-intron analysis were generated using the online program GSDS^7^.

### Subcellular Localization of CaBiP1 Protein

The *CaBiP-1* ORF without a termination codon was amplified using specific primer pair GFP-CaBiP1-F and GFP-CaBiP1-R (Supplementary Table S1) from a cDNA template isolated from R9 leaf material grown under normal growth conditions, and then the PCR product was cloned into the pBI221 expression vector containing green GFP. An empty vector without *CaBiP-1* was used as the control. Particle bombardment was performed to introduce recombinant plasmid into onion epidermal cells. ER-Tracker Red (Beyotime, C1041, China), a specific fluorescent probe for ER, was used to highlight this cellular component. Details of the methods can be found in our previous study (Guo et al., 2014).

### Virus-Induced Gene Silencing (VIGS) of *CaBiP1*

A 346 bp fragment of the *CaBiP1* ORF was amplified by gene-specific primer pair TRV2-CaBiP1-F and TRV2-CaBiP1-R (Supplementary Table S1) from a cDNA template isolated from R9 leaf material grown under normal growth conditions, and the PCR product was cloned into the pMD19T vector (Takara, Dalian, China). After digestion with restriction enzymes of *Xba* I and *Kpn* I, the *CaBiP1* fragment was cloned into the pTRV2 virus expression vector to generate the TRV2:*CaBiP1* silencing construct. The empty TRV2:00 vector without *CaBiP1* was used as the control, and TRV2:*CaPDS* (phytoene desaturase gene) was used as a marker for gene silencing. *Agrobacterium tumefaciens* strain GV3101 cells containing TRV2:*CaBiP1*, TRV2:00 or TRV2:*CaPDS* were separately injected into the leaves of the R9 thermotolerant pepper line as described by Wang et al. (2013). When the photo-bleaching phenotype was evident in pepper seedlings carrying TRV2:*CaPDS*, the silencing efficiency of pTRV2:*CaBiP1* was assessed by qRT-PCR with the primer pair of qCaBiP1-F and qCaBiP1-R (Supplementary Table S1).

### Generation of *Arabidopsis* Lines Overexpressing *CaBiP1*

The full-length *CaBiP1* ORF was amplified from cDNA isolated from R9 leaf material grown under normal growth conditions with the gene-specific primer pair of CaBiP1-F and CaBiP1-R (Supplementary Table S1). The amplification product was inserted into the plant transformation binary vector pBI121 between the CaMV-35S promoter and the *nos* (nopaline synthase) terminator. The resultant pBI121 vector was transformed into *Arabidopsis* ecotype Col-0 using the floral dip method intermediated by *Agrobacterium* GV3101 (Clough and Bent, 1998). Transgenic plants were obtained by screening successive generations for kanamycin resistance, and T3 seeds were used for subsequent experiments.

### Experimental Treatments and Samples Collection

For tissue-specific expression analysis of *CaBiP* genes, young leaves, flower buds, fruits (about 1 cm in length), stems and roots were collected from pepper plants grown under normal conditions. Seedlings at the six-leaf stage were used for abiotic stress treatments. For abiotic stress treatments involving abscisic acid (ABA), H_2_O_2_, DTT, the plants were sprayed with 0.1 mM ABA, 1 mM H_2_O_2_, or 15 mM DTT until leaves were thoroughly wetted, and leaves were collected at 0, 1, 3, 6, 12, and 24 h post treatment. For salt and osmotic stress experiments, the roots of the seedling were soaked in 200 mM NaCl and 200 g⋅L^-1^ PEG6000, respectively, and leaves and roots were sampled at 0, 1, 3, 6, 12, and 24 h post treatment. For heat treatment, pepper seedlings were incubated at 45°C, and leaves were collected at 0, 0.5, 1, 2, 4, and 6 h post treatment. All samples were immediately frozen in liquid nitrogen and kept at -80°C for RNA extraction.

Pepper seedlings of TRV2:*CaBiP1* and TRV2:00 were used for abiotic stress treatments. For ER stress, pepper seedlings were sprayed with 30 mM DTT until leaves were thoroughly wetted and incubated for 24 h. For heat stress, pepper seedlings were exposed to 45°C for 24 h then allowed to recover for 5 days under normal conditions. For osmotic stress, the seedlings were soaked in 300 g⋅L^-1^ PEG for 24 h. For drought stress, pepper seedlings were deprived of water for 10 days, after which the RWC was determined. For dehydration tests, detached leaves were placed on the bench and weighted from 0 to 240 min at intervals of 30 min. For salt stress, pepper seedlings were divided into two groups, and one group was irrigated with 300 mM NaCl for 14 days, while the root of the other group was soaked in 300 mM NaCl for 24 h. After treatment, pepper leaves were sampled immediately used for determination of H_2_O_2_, REL, MDA, and soluble protein.

Seeds from *Arabidopsis* overexpressing *CaBiP1* and wild-type Col-0 were germinated on MS plates with 3 mM DTT for ER stress, and 5-day-old seedlings grown on normal medium were transferred to medium with 2 mM DTT for 15 days. For heat stress, the MS plates with 7-day-old transgenic *Arabidopsis* seedlings were immersed in a water bath at 45°C for 50 min, then recovered at 22°C for 5 days, while 2-week-old seedlings in pots were heat-treated at 45°C for 6 h and recovered at 22°C for 7 days in a controlled temperature chamber. For salt and osmotic stress tests, *Arabidopsis* seeds were grown on MS medium with 100 mM NaCl or 200 mM mannitol for 4 days to determine the germination rate, and on medium with 75 mM NaCl or 150 mM mannitol for 7 days to determine root length. For salt stress experiments, 2-week-old seedlings were watered with 300 mM NaCl for 15 days to determine the survival rate. For dehydration tests, the detached *Arabidopsis* leaves were placed on the bench and weighted from 0 to 240 min at intervals of 30 min. *Arabidopsis* seedlings were deprived of water for 12 days to measure the RWC, root length, and re-watered for 2 days to determine the survival rate. In addition, 3-week-old *Arabidopsis* seedlings were soaked with root in 300 mM NaCl or 300 mM mannitol solutions, or sprayed with 30 mM DTT, or exposed to high temperatures of 45°C, and leaves were collected at 0, 3, and 9 h post treatments for genes expression analysis. For drought stress, 2-week-old *Arabidopsis* seedlings were withheld water for 10 days for gene expression analysis. All experiments were performed with three biological replicates.

### Measurement of REL, H_2_O_2_, MDA, RWC, and Soluble Protein

Pepper leaf discs were used to measure the REL according to the method of Yin et al. (2014), and the MDA content was determined using the thiobarbituric acid reaction according to Dhindsa and Thorpe (1981). H_2_O_2_ levels were assessed by the DAB staining method (Dang et al., 2013), and the soluble protein content was measured by the protein dye-binding method with Coomassie light blue (Bradford, 1976). RWC was measured as described by Chakraborty et al. (2012). All experiments were performed with three biological replicates.

### Total RNA Extraction, cDNA Synthesis, and qRT-PCR Analysis

Total RNA was extracted from sampled plant tissues using the Trizol (Invitrogen, Carlsbad, CA, United States) method, and the residual genomic DNA was digested by RNase-free DNase I (Promega, Madison, WI, United States). The first-strand cDNA was synthesized using the PrimeScript^TM^ Kit according to the manufacturer’s instructions (TaKaRa, Tokyo, Japan). Primer pairs (Supplementary Table S1) were designed by NCBI Primer-BLAST^8^, and qRT-PCR was performed using SYBR^®^ Premix Ex Taq^TM^ II (TaKaRa) as described previously by Wang et al. (2013). Relative gene expression levels were analyzed according to the 2^-ΔΔCT^ method (Livak and Schmittgen, 2001), and *CaUBI3* and *AtActin2* were used as internal controls in pepper and *Arabidopsis*, respectively. Significance tests for differences in gene expression levels between control and stress treatments were performed using the Student’s *t*-test method at the α = 0.05 and 0.01 levels.

## Results

### Sequence Analysis of CaBiP Proteins

**FIGURE 1:**
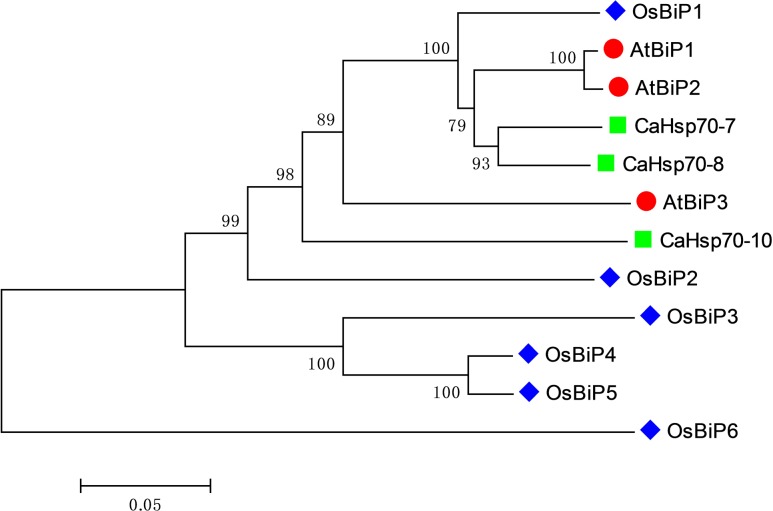
Phylogenetic tree of BiP family members from representative plant species. Ca, *Capsicum annuum L.*; At, *Arabidopsis thaliana*; Os, *Oryza sativa*.

The phylogenetic tree of BiP proteins from pepper, *Arabidopsis* and rice showed that CaHsp70-8 and CaHsp70-7 share a close evolutionary relationship with AtBiP1 and AtBiP2, respectively, while CaHsp70-10 is more closely related to AtBiP3 and OsBiP2 (**Figure 1**). Therefore, CaHsp70-8, CaHsp70-7, and CaHsp70-10 were renamed as CaBiP1, CaBiP2, and CaBiP3, respectively.

**FIGURE 2:**
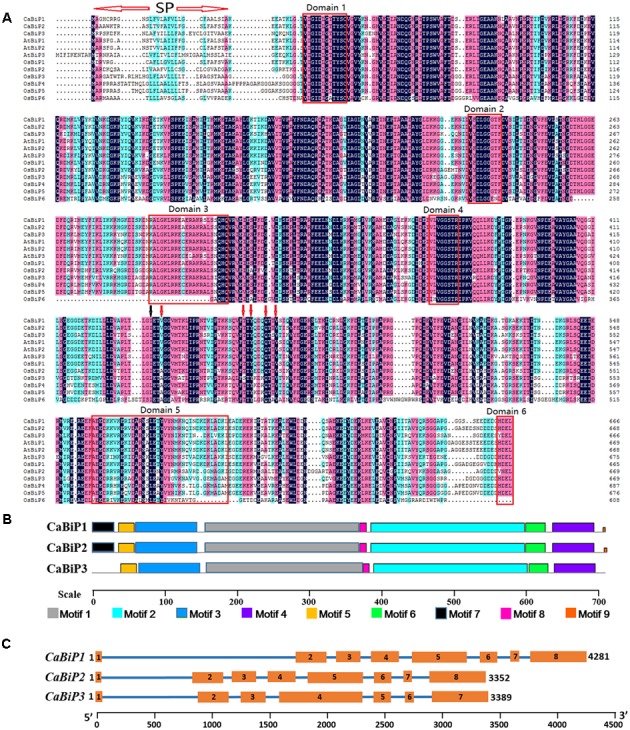
Sequence analysis of *CaBiP* genes. **(A)** Conserved amino acid domains in CaBiPs shown by amino acid sequence alignment with orthologs in *Arabidopsis* and rice. SP, the signal peptide. Red arrows indicate hydrogen bonds, black arrow indicates the the cut-off point GI. **(B)** Motifs of CaBiP proteins identified by MEME tools. Different motifs are indicated by different colors. **(C)** Distribution of introns and extrons in *CaBiP* genes. Blue lines = introns, orange rectangles = extrons.

Alignment of CaBiP protein sequences with AtBiPs (AtBiP1, NP_198206; AtBiP2, NP_851119; AtBiP3, NP_172382) and OsBiPs (OsBiP1, XP_015625618; OsBiP2, XP_015629631; OsBiP3, XP_015638605; OsBiP4, XP_015638801; OsBiP5, BAT04227; OsBiP6, LOC_Os01g33360) revealed high conservation in the signaling and functional domains. Seven domains and nine motifs were identified in CaBiPs based on published *Arabidopsis* and rice literatures (Noh et al., 2003; Sarkar et al., 2013) (**Figures 2A,B**). A signal peptide sequence (SP) for membrane transport was detected at the beginning of the *N*-terminus in CaBiPs, and the cut-off point GI (labeled by black arrow), a conserved site separating ATPase domain from the peptide-binding domain, was also found. In the *N*-terminal ATPase region, all the essential domains for BiP ATPase activity, including Domain 1 (motif 5) and Domain 2 (*N*-terminus of motif 1) for phosphate-binding, and Domain 4 (motif 2) for adenosine binding, are conserved in the predicted CaBiP proteins. Meanwhile, Domain 3 (*C*-terminus of motif 1) for calmodulin binding was also identified in CaBiPs. The *C*-terminal protein binding region has a highly conserved five residue core (shown by red arrows) that mediates hydrogen bonding with peptide substrates. However, Domain 5 (motif 6), which is essential for binding to peptide substrates, differs between CaBiP3 and CaBiP1/CaBiP2. Although CaBiP3 has the same pattern in motif distribution pattern found in CaBiP1 and CaBiP2, motif 7 containing the SP and motif 9 containing the ER retention signal are absent in CaBiP3 (**Figure 2B** and Supplementary Table S2). Analysis of gene structure also revealed that CaBiP3 lacks an intron that is present in CaBiP1 and CaBiP2 (**Figure 2C**).

### Expression Profiles of *CaBiP* Genes in Pepper Tissues

**FIGURE 3:**
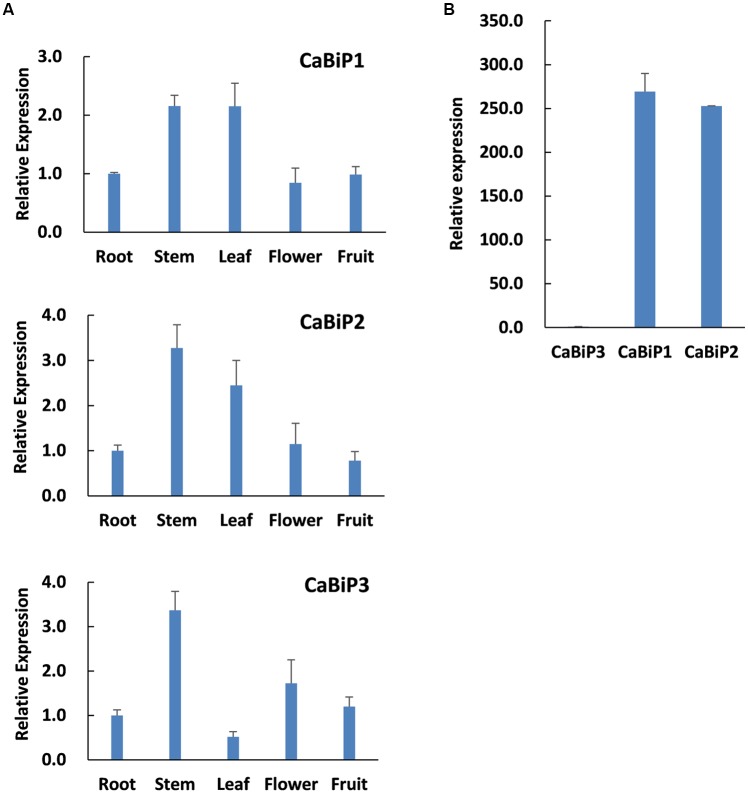
Tissue-specific expression *CaBiP genes*. **(A)** Expression of *CaBiP1*, *CaBiP2* and *CaBiP3* in pepper root, stem, leaf, flower and fruit. **(B)** Relative expression of *CaBiP* genes in pepper root. The expression level of *CaUBI3* was used as the internal control. Bars shows the standard deviation of expression levels from three biological replications.

Expression profiles of the three *CaBiP* genes in pepper root, stem, leaf, flower and fruit tissues were determined by qRT-PCR with transcript-specific primers (Supplementary Table S1). The results indicated that the three *CaBiP* genes were diversely expressed in roots, stems, leaves, flowers, and fruits (**Figure 3A**), and the overall expression level of *CaBiP3* was much lower than *CaBiP1* and *CaBiP2* (**Figure 3B**). In addition, the expression levels of *CaBiP1* and *CaBiP2* genes were obviously higher in stem and leaf tissue than in other organs, while *CaBiP3* expression was higher in stem and flower (**Figure 3A**).

### Expression of *CaBiP* Genes in Pepper Under Abiotic Stresses in Pepper

**FIGURE 4:**
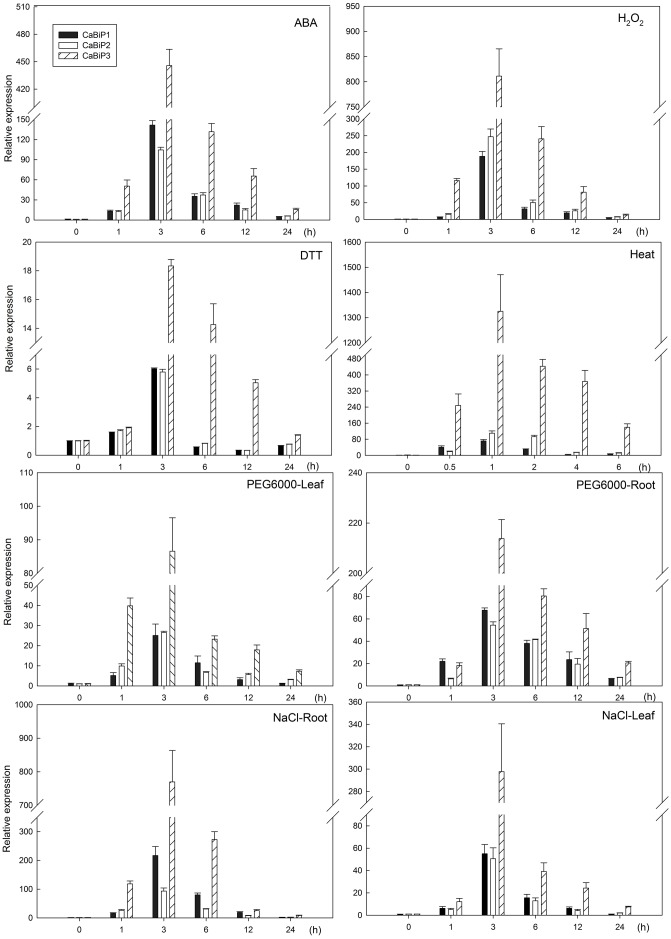
Expression of *CaBiP* genes following salt, osmotic, heat, ER, ABA, and H_2_O_2_ stress treatments. Bars show the standard deviation of expression levels from three biological replications.

Expression of all *CaBiP* genes was induced under abiotic stress conditions involving ABA, H_2_O_2_, DTT, heat, salt, and osmotic stresses (**Figure 4**). Peak expression occurred earlier in heat stress (1 h) than in other treatments (3 h), and the fold change in *CaBiP3* expression was significantly higher than that of *CaBiP1* and *CaBiP2* in all treatments. In addition, *CaBiP* was expressed more highly in roots than in leaves under osmotic stress and salt stress.

### Subcellular Localization of CaBiP1 in the ER

**FIGURE 5:**
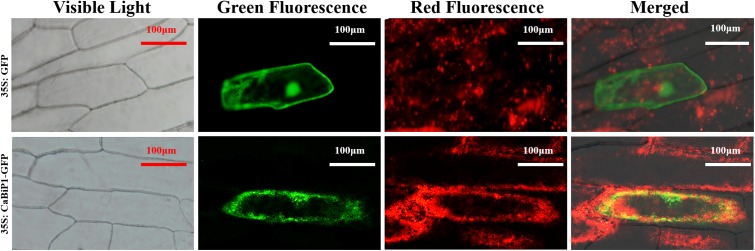
Subcellular localization of the CaBiP1-GFP fusion protein in onion epidermal cells. CaMV35S:GFP was used as the control, and ER-Tracker Red was recruited to mark the ER. CaMV35S, constitutive promoter from the cauliflower mosaic virus. GFP, Bars = 100 μm. More than 10 transgenic cells were observed, and all of them had the similar result. One of them was presented here.

Under the control of CaMV-35S promoter, the 35S:CaBiP1-GFP construct was transiently expressed in onion epidermal cells. While both the green fluorescence from fusion protein of CaBiP1 and GFP and the red fluorescence from ER-Tracker Red were detected by laser confocal microscopy, their merged yellow fluorescence was also observed (**Figure 5**). The results indicate CaBiP1 is located in the ER.

### ER Stress Tolerance Is Altered in *CaBiP1-*Silenced and *CaBiP1*-Overexpressing Plants

Pepper seedlings harboring TRV2:*CaBiP1* demonstrated a silencing efficiency of over 70%, and *Arabidopsis* transgenic lines OE7 and OE8 were used to perform abiotic stress treatments. No visible difference was observed between either TRV2:*CaBiP1* and TRV2:00 pepper lines, and the same was true for *CaBiP1*-OE lines and WT *Arabidopsis* plants (**Supplementary Figure S1**).

**FIGURE 6:**
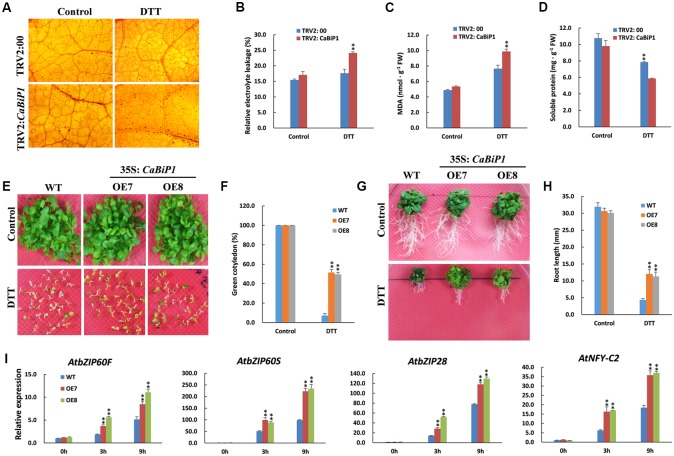
Altered ER stress tolerance in *CaBiP1*-silenced and *CaBiP1*-overexpressing plants. **(A–D)** H_2_O_2_ accumulation, REL, MDA content, and soluble protein content in leaves of TRV2:00 and TRV2:*CaBiP1* pepper seedlings grown under ER stress induced by spraying 30 mM DTT for 24 h. Spraying with distilled water was performed on control plants. **(E,F)** Establishment of WT and *CaBiP1-*OE *Arabidopsis* seedling under ER stress conditions induced by 3 mM DTT. The percentage of seedlings with green cotyledons was calculated at the14th day after sowing. **(G,H)** Root growth of 5-day-old WT and transgenic *Arabidopsis* seedlings exposed to 2 mM DTT for 15 days. **(I)** Expression analysis of ER stress-responsive genes in WT and *CaBiP1*-OE lines sprayed with 30 mM DTT for the designed time duration. *AtActin2* was used as the internal control. Data are the mean ± standard deviation from three biological replicates. Statistical significance is indicated by a single asterisk (*p* < 0.05) and a double asterisk (*p* < 0.01) based on the results of the Student’s *t*-test.

Following ER stress induced by 30 mM DTT, the H_2_O_2_ content in pepper leaves, as revealed by dark brown dots after DAB staining, was increased in both TRV2:*CaBiP1* and TRV2:00 lines, albeit to a greater extent in the former (**Figure 6A**). Similar results were also observed for REL levels and MDA content (**Figures 6B,C**). By contrast, the soluble protein content was decreased in the leaves of both TRV2:*CaBiP1* and TRV2:00 plants following ER stress, and the decrease was again more significant in the former (**Figure 6D**).

Under normal growth conditions, no difference was observed in the germination rate between *CaBiP1*-OE lines and WT *Arabidopsis* lines. However, under ER stress induced by 3 mM DTT, the germination rate was significantly higher in *CaBiP1*-OE plants (**Supplementary Figure S2A**). Furthermore, when the treatment was extended to 14 days, chlorosis of cotyledons was more severe in WT than in *CaBiP1*-OE lines (**Figures 6E,F**). After *Arabidopsis* seedlings at 5-day-old were transferred to MS medium containing 2 mM DTT, the growth was inhibited in both *CaBiP1*-OE and WT lines, but fresh weight and root length were obviously higher in the *CaBiP1*-OE plants (**Figures 6G,H** and **Supplementary Figure S2B**). Additionally, expression of some genes in the UPR pathway, such as *AtbZIP60F*, *AtbZIP60S*, *AtbZIP28*, and *AtNF-YC2* (encoding a component of the *AtbZIP28* transcription complex; Liu and Howell, 2010), were up-regulated when the *Arabidopsis* seedlings were exposed to DTT, and the fold change was greater in transgenic lines than in WT plants (**Figure 6I**).

### Heat Tolerance Is Altered in *CaBiP1-*Silenced and *CaBiP1*-Overexpressing Plants

**FIGURE 7:**
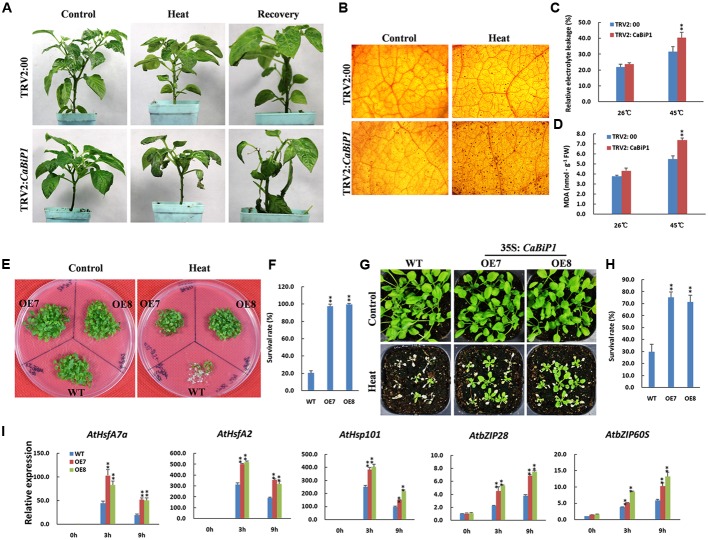
Altered heat tolerance in *CaBiP1*-silenced and *CaBiP1*-overexpressing plants. **(A)** TRV2:*CaBiP1* and TRV2:00 pepper seedlings following heat stress at 45°C for 24 h and recovery for 5 days under 25°C normal conditions. Plants continually grown at 25°C were used as controls. **(B–D)** H_2_O_2_ accumulation,REL and MDA content in pepper seedlings leaves treated with 45°C heat stress for 24 h. **(E,F)** Survival rate of 7-day-old WT and *CaBiP1*-OE *Arabidopsis* seedlings submerged in a 45°C water bath for 50 min and recovered at 22°C for 5 days. **(G,H)** Survival rate of 2-week-old *Arabidopsis* seedlings in soil after treatment at 45°C for 6 h then recovered for 5 days. **(I)** Expression of ER stress- and heat stress-related genes in 3-week-old WT and *CaBiP1*-OE *Arabidopsis* seedlings treated at 45°C for the designed time duration. *AtActin2* was used as the internal control. Data are the mean ± standard deviation from three biological replicates. Statistical significance is indicated by a single asterisk (*p* < 0.05) and a double asterisk (*p* < 0.01) based on the results of the student’s *t*-test.

When TRV2:*CaBiP1* and TRV2:00 pepper seedlings were subjected to 45°C for 24 h, sunburn-like symptoms of heat stress was observed in TRV2:*CaBiP1* leaves but not in those of TRV2:00 plants. After recovery under normal conditions for 5 days, the injured leaves in TRV2:*CaBiP1* fell off, while TRV2:00 leaves resumed growth (**Figure 7A**). Under heat stress, H_2_O_2_, MDA content, and REL levels were increased in both TRV2:*CaBiP1* and TRV2:00 lines, and all three indicators were markedly higher in the former (**Figures 7B–D**).

*Arabidopsis* seedlings of 1-week-old were treated at 45°C for 50 min, then recovered for 5 days under normal conditions, and only 20% of WT plants survived, compared with more than 90% of *CaBiP1-*OE plants (**Figures 7E,F**). When 2-week-old seedlings of WT and *CaBiP1*-OE lines in pots were subjected to 45°C for 6 h then recovered for 5 days, about 30% of WT plants survived, compared with 74.1% for OE7 and 70.4% for OE8 plants (**Figures 7G,H**). During heat stress treatments, both UPR-related genes (*AtbZIP60S* and *AtbZIP28*) and heat responsive genes (*AtHsfA2, AtHsfA7a*, and *AtHsp101*) were obviously induced in both WT and transgenic *Arabidopsis* lines. However, the induction of gene expression in transgenic lines was more intense than in WT plants. Furthermore, the expression levels of three heat response genes peaked at 3 h, while those of two UPR genes increased continuously during the treatments (**Figure 7I**).

### Salt Tolerance Is Altered in *CaBiP1-*Silenced and *CaBiP1*-Overexpressing Plants

**FIGURE 8:**
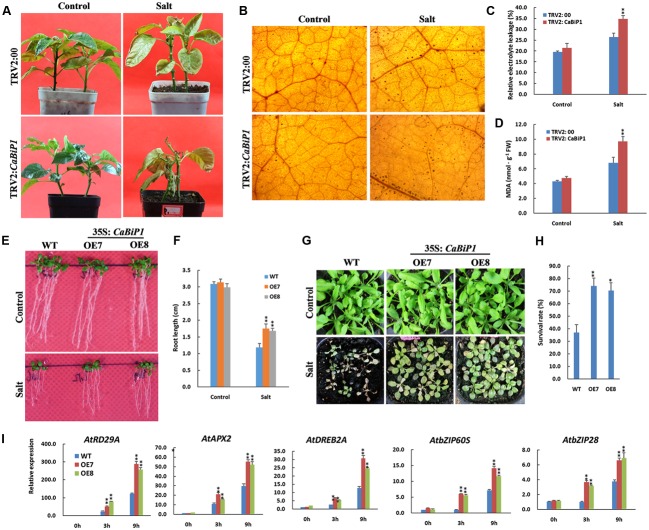
Altered salt tolerance in *CaBiP1*-silenced and *CaBiP1*–overexpressing plants. **(A)** TRV2:00 and TRV2:*CaBiP1* peppers seedlings irrigated with 300 mM NaCl solution for 14 days. **(B–D)** H_2_O_2_ accumulation, REL and MDA content in pepper seedlings leaves treated with 300 mM NaCl solution for 24 h. **(E,F)** Root growth of WT and transgenic lines grown under 75 mM NaCl for 7 days. **(G,H)** Survival rate of 2-week-old *Arabidopsis* seedlings in soil treated with 300 mM NaCl for 15 days. **(I)** Expression of salt stress- and ER stress-related genes in leaves of 3-week-old WT and *CaBiP1*-OE hydroponic seedlings treated with 300 mM NaCl s for the designed time duration. *AtActin2* was used as the internal control gene. Data are the mean ± standard deviation from three biological replicates. Statistical significance is indicated by a single asterisk (*p* < 0.05) and a double asterisk (*p* < 0.01) based on the results of student’s *t*-test.

After irrigation with 300 mM NaCl solution for 14 days, the leaves of *CaBiP1*-silenced pepper seedlings became yellowish and wilted and eventually fell off, while in TRV2:00 plants, only yellowness was observed (**Figure 8A**). When the root of TRV2:*CaBiP1* and TRV2:00 seedlings were soaked in 300 mM salt solution for 24 h, the accumulation of H_2_O_2_ in TRV2:*CaBiP1* leaves was higher than in TRV2:00 leaves (**Figure 8B**), and the MDA content was also 1.4-fold higher in TRV2:*CaBiP1* leaves (**Figure 8C**). Similarly, REL levels were higher in TRV2:*CaBiP1* (36.8%) than TRV2:00 (26.4%) (**Figure 8D**).

When *Arabidopsis* seedlings were grown on MS medium with 100 mM NaCl for 4 days, the germination rate of *CaBiP1*-OE lines was obviously higher than that of WT plants (**Supplementary Figure S3**). Additionally, in *Arabidopsis* seedlings grown on MS plates containing 75 mM NaCl for 7 days, the roots of *CaBiP1*-OE lines were significantly longer than those of WT plants (**Figures 8E,F**). Following watering of 2-week-old seedlings with a high concentration of NaCl (300 mM) for 15 days, *CaBiP1*-OE seedlings showed a higher salt tolerance than WT plants; the survival rates of *CaBiP1*-OE7 and OE8 plants were 2.0- and 1.9-fold higher than WT, respectively (**Figures 8G,H**). In terms of gene expression, salt stress-related genes (*AtAPX2*, *AtDREB2A*, and *AtRD29A*) and UPR-related genes (*AtbZIP60S* and *AtbZIP28*) displayed higher levels of transcription in *CaBiP1*-OE plants than in WT plants (**Figure 8I**). Interestingly, the expression of all the tested genes revealed continuous enhancement throughout the duration of salt stress experiments (**Figure 8I**).

### Osmotic Stress Tolerance Is Altered in *CaBiP1-*Silenced and *CaBiP1*-Overexpressing Plants

**FIGURE 9:**
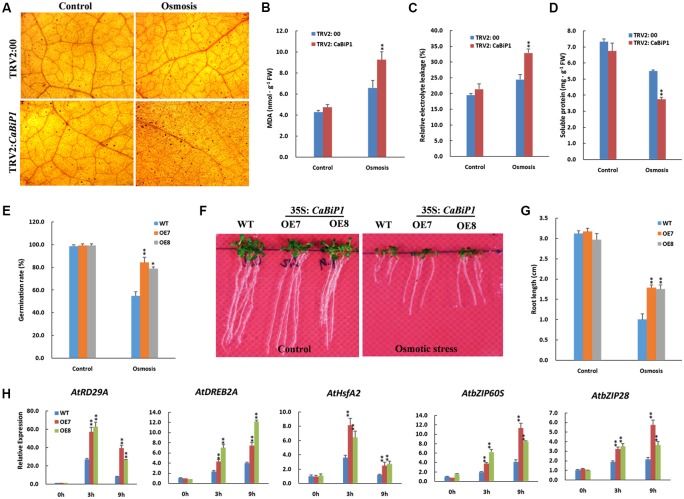
Altered osmotic tolerance in *CaBiP1*-silenced and *CaBiP1*-overexpressing plants. **(A–D)** H_2_O_2_ accumulation, REL, MDA content, and soluble protein content in TRV2:00 and TRV2:*CaBiP1* pepper seedlings leaves grown under osmotic stress simulated by soaking in 300 g/L PEG6000 for 24 h. Treatment with distilled water was used as the control. **(E)** Seed germination rate of WT and *CaBiP1-*OE lines grown under osmotic stress by adding 200 mM mannitol in the medium for 4 days. **(F,G)** Root growth of WT and transgenic *Arabidopsis* lines germinated under 150 mM mannitol for 7 days. **(H)** Expression of osmotic stress- and ER stress-related genes in the leaves of 3-week-old WT and *CaBiP1*-OE hydroponic seedling grown under 300 mM mannitol for designed time duration. *AtActin2* was used as the internal control gene. Data are the mean ± standard deviation from three biological replicates. Statistical significance is indicated by a single asterisk (*p* < 0.05) and a double asterisk (*p* < 0.01) based on the results of the Student’s *t*-test.

After soaking in 300 g⋅L^-1^ PEG6000 solution for 24 h, TRV2:*CaBiP1* pepper seedlings accumulated more H_2_O_2_ than did TRV2:00 plants, as shown by the dark brown dots in leaves after DAB staining (**Figure 9A**). Similarly, the MDA content and REL levels in TRV2:*CaBiP1* seedlings were obviously higher than those of TRV2:00 lines following osmotic stress (**Figures 9B,C**). By contrast, the soluble protein content in TRV2:*CaBiP1* leaves was significantly lower than in TRV2:00 leaves (**Figure 9D**). Following osmotic stress, *Arabidopsis* seeds from *CaBiP1*-OE plants grown on MS medium containing 200 mM mannitol displayed a remarkably high germination rate compared with that of WT plants; seed germination in OE7 and OE8 lines reached to 84 and 78% at the 4th day, respectively, whereas that of WT was only 54% (**Figure 9E**). On the 7th day after germination on MS medium containing 150 mM mannitol, *CaBiP1*-OE *Arabidopsis* seedlings exhibited stronger tolerance to osmotic stress, as evidenced by a 1.8-fold increase in root length compared with WT plants (**Figures 9F,G**). The qRT-PCR analysis indicated that osmotic stress-related genes (*AtDREB2A, AtRD29A*, and *AtHsfA2*) were highly induced in stressed leaves (**Figure 9H**). Notably, *AtRD29A*, a marker for osmotic stress, as up-regulated by 5.0- and 3.3-fold at 9 h in OE7 and OE8 lines compared with WT plants. Similarly, *CaBiP1*-OE lines showed higher expression levels of ER stress-related genes (*AtbZIP60S* and *AtbZIP28*) at 3 h and 9 h following exposure to 300 mM mannitol. Additionally, expression of *AtRD29A* and *AtHsfA2* peaked at 3 h, while expression of the other three genes increased continuously throughout the treatment (**Figure 9H**).

### Drought/Dehydration Tolerance Is Altered in *CaBiP1-*Silenced and *CaBiP1*-Overexpressing Plants

**FIGURE 10:**
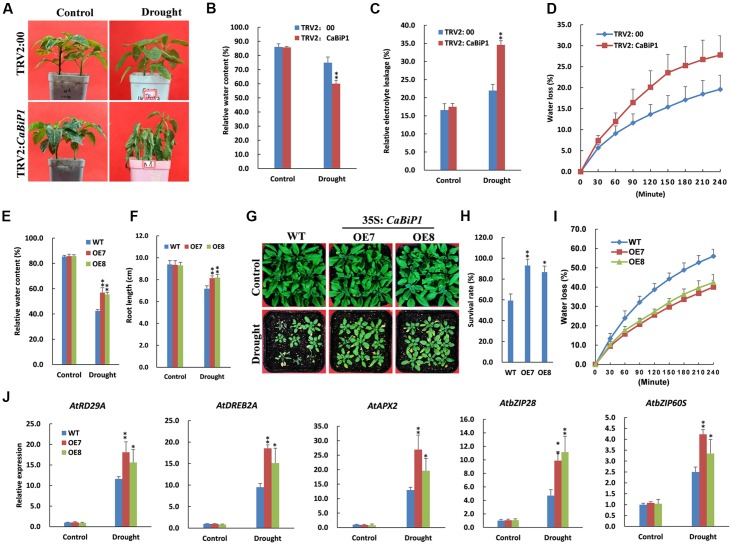
Altered drought tolerance in *CaBiP1*-silenced and *CaBiP1*-overexpressing plants. **(A–C)** Symptoms, RWC and REL in the leaves of TRV2:*CaBiP1* and TRV2:00 pepper seedlings grown under drought stress performed by withholding water for 10 days. **(D)** Water loss rate of detached leaves from pepper TRV2:*CaBiP1* and TRV2:00 seedlings. **(E,F)** RWC and root length of WT and *CaBiP1*-OE lines grown under drought stress by withholding water for 12 days. **(G,H)** Survival rate of WT and *CaBiP1*-OE lines grown under drought stress by withholding water for 12 days then rehydrated for 2 days. **(I)** Water loss rate of detached leaves from WT and *CaBiP1*-OE seedlings. **(J)** Expression of drought stress- and ER stress-related genes in the leaves of WT and *CaBiP1*-OE lines grown under drought stress by withholding water for 10 days. *AtActin2* was used as the internal control gene. Data are the mean ± standard deviation from three biological replicates. Statistical significance is indicated by a single asterisk (*p* < 0.05) and a double asterisk (*p* < 0.01) based on the Student’s *t*-test.

At the end of the drought stress treatment that involved withholding water for 10 days, wilting symptoms were observed in pepper leaves of TRV2:*CaBiP1* lines but not TRV2:00 (**Figure 10A**). Furthermore, TRV2:*CaBiP1* plants exhibited higher REL level and lower RWC than TRV2:00 (**Figures 10B,C**). Similarly, dehydration of pepper leaves placed on a bench for 4 h resulted in a rate of water loss that was higher in gene-silenced pepper seedlings compared with controls (**Figure 10D**).

Following the withholding of water for 12 days, *Arabidopsis* seedlings became severely withered, but *CaBiP1*-OE seedlings exhibited higher RWC and longer root than WT plants (**Figures 10E,F**). After re-watering for 2 days, more than 90% of *CaBiP1*-OE *Arabidopsis* seedlings survived, whereas just 59% WT plants remained alive (**Figures 10G,H**). In addition, under dehydration conditions, the rate of water loss for detached rosette leaves from transgenic seedlings was markedly lower than that in WT leaves (**Figure 10I**). Additionally, *CaBiP1*-OE lines showed a more obvious induction in the drought stress-related genes (*AtDREB2A, AtRD29A*, and *AtAPX2*) and ER stress-related genes (*AtbZIP60S* and *AtbZIP28*) under drought stress than did WT plants (**Figure 10J**).

## Discussion

A major negative effect of abiotic stress on plant cells is the accumulation of misfolded and denatured proteins, which further induces ER stress. The alleviation of ER stress is therefore key to plant survival under adverse environmental conditions (Wan and Jiang, 2016). BiP, an Hsp70 family member located in the ER, binds to nascent proteins to protect them from aggregation (Howell, 2013). Characterization and functional analysis of BiPs will therefore contribute to our understanding of plant tolerance to abiotic stress.

In our previous study, three BiP proteins (CaBiP1, CaBiP2, and CaBiP3) were identified in the genome of pepper (Guo et al., 2016). Amino acid sequence alignment revealed the conserved functional and signaling domains in CaBiPs, including an ATP-binding domain at the *N*-terminus (Domain 1, 2, and 4), a protein-binding domain at the *C*-terminus (Domain 5), and an ER retention factor (Domain 6) (**Figure 2A**) (Noh et al., 2003; Sarkar et al., 2013), indicating the importance of the different domains and ER localization of CaBiP1 (**Figure 5**).

CaBiP1 and CaBiP2 share high sequence similarity, whereas CaBiP3 is more distantly related, with diversity particularly high in Domain 5 (motif 6), Domain 6, motif 7, and motif 9 (**Figures 2A,B**), and difference in the exon-intron structure (**Figure 2C**). Correspondingly, differences in expression pattern were also observed between *CaBiP3* and *CaBiP1*/*CaBiP2*. While *CaBiP1* and *CaBiP2* were constitutively expressed in all tested tissues (**Figure 3**), transcription of *CaBiP3* was barely detectable under normal growth conditions, but expression was strongly induced by ER stress and various environmental stresses such as heat, osmotic and salt stress, and so by exposure to the signaling molecules such as ABA and H_2_O_2_ (**Figure 4**). Similar phenomena were also found in *Arabidopsis* and rice, in which *AtBiP1*, *AtBiP2* and *OsBiP1* were constitutively expressed in various tissues, whereas mRNA levels of *AtBiP3*, *OsBiP4*, and *OsBiP5* remained low under normal conditions but were strongly up-regulated by ER stress agents of TM or DTT (Noh et al., 2003; Wakasa et al., 2012).

In agreement with our results, Noh et al. (2003) found that *AtBiP3* was distinguishable from *AtBiP1* and *AtBiP2* in several ways, including the regulatory elements in the promoter region, genomic organization, and sequence homology. Furthermore, Wakasa et al. (2012) also suggested that OsBiP4 and OsBiP5 may have a function that is distinct from that of OsBiP1. Guo et al. (2016) revealed that the *cis*-elements in *CaBiP3* were significantly different from those in *CaBiP1* and *CaBiP2*. These results indicate a relationship between the gene structures of *CaBiPs* and their expression patterns, and even biological functions, but further research is needed.

The contribution of *CaBiP1* in plant tolerance to environmental stress was revealed by VIGS experiments in pepper and overexpression experiments in *Arabidopsis*. Our results suggest that the silencing of *CaBiP1* decreased the tolerance of pepper to multiple abiotic stresses, including heat, osmotic, salt, drought/dehydration and ER stress, and conversely, overexpression of *CaBiP1* increased the tolerance of *Arabidopsis* to these stresses. Under stress conditions, the *CaBiP1*-silenced pepper line presented more severe injury symptoms based on various physiological indicators, while *CaBiP1*-overexpressing *Arabidopsis* lines displayed higher tolerance to adverse environments in terms of seed germination rate, seedling survival rate, and root length (**Figures 6**–**10**). Similar results were also found following ER and heat stresses in *Arabidopsis* (Yang et al., 2016), and following water deficit in tobacco (Alvim et al., 2001) and pathogen infection experiments in tomato (Liebrand et al., 2014). These results suggest that *CaBiP1* confers plant enhanced tolerance to multiple environmental stress factors.

Abiotic stress induces the production of ROS such as H_2_O_2_, a secondary messenger that regulates protective mechanism of plant cells. However, excess ROS causes irreversible damage leading to cell death (Ozgur et al., 2014). After treatment with DTT and following abiotic stress, the accumulation of ROS was higher in *CaBiP1*-silenced pepper leaves than in WT plants, and the MDA content and REL were consequently elevated (**Figures 6**–**10**). These results suggest that down-regulation of the *CaBiP1* gene worsens the cell injuries raised by adverse environmental conditions that cause accumulation of ROS. In accordance with our results, Alvim et al. (2001) also found that antisense silencing of the *BiP* gene diminishes water deficit-induced oxidative stress in tobacco, which is characterized by increased superoxide dismutase activity.

In addition, under ER and osmotic stress conditions, the soluble protein content in *CaBiP1*-silenced pepper leaves was significantly lower than in the WT line (**Figures 6**, **9**), consistent with the study by Yang et al. (2016), in which overexpression of *AtBiP1* alleviated autophagy pressure in *Arabidopsis* and decreased protein degradation following heat stress. These results indicate that *CaBiP1* may protect plant cells from environmental stress by preventing protein aggregation. *CaBiP1*-OE *Arabidopsis* plants exhibited slower water loss in detached rosette leaves, but higher RWC than WT plants under water deficit conditions (**Figures 10C,D**). Conversely, *CaBiP1*-silenced pepper leaves showed faster water loss, but lower RWC than control plants under water deficit conditions (**Figures 10E,I**). Similarly, when the soybean *BiP* gene was overexpressed in soybean and tobacco, transgenic lines were better able to maintain cellular homeostasis under water stress conditions, and displayed higher leaf water content and reduced withering (Alvim et al., 2001; Valente et al., 2009; Carvalho et al., 2014a). Since water deficit can be caused by other abiotic stress such as heat, salt and osmotic stress, these results indicate that *CaBiP1* confers plant tolerance to adverse environmental conditions by increasing water retention capacity of the cells.

When *Arabidopsis* seedlings were exposed to ER stress induced by DTT, transcription of UPR genes *bZIP28* and *bZIP60S* was continuously increased throughout the treatment (**Figure 6**). Similarly, expression of UPR genes was also continuously enhanced under the duration of various abiotic stresses (**Figures 7**–**10**). Based on these combined results in *Arabidopsis*, it appears ER stress is always concomitant with abiotic stress, and alleviation of ER stress is therefore conducive to improving plant tolerance to adverse environments. Furthermore, under abiotic stress treatments, expression of *AtbZIP28* and *AtbZIP60S* in *CaBiP1*-overexpressing *Arabidops*is lines was markedly higher than in WT plants (**Figures 7**–**10**). Since AtbZIP28 and AtbZIP60 are transcription factors involved in up-regulating protective genes in different branches of the UPR pathway (Howell, 2013), these results suggest overexpression of *CaBiP1* confers higher efficiency of the protein folding machinery under stress conditions. However, Leborgne-Castel et al. (1999) reported that overexpression of *BiP* in tobacco decreased the UPR pathway, and up-regulation of BiP in the transgenic line was sufficiently elevated to assist protein folding during ER stress. Based on the role of BiP as a sensor in ER stress signaling (Bertolotti et al., 2000; Shen et al., 2002), we believe CaBiP1 performs protective functions as both a molecular chaperone and UPR regulator, consistent with research on *Arabidopsis* by Srivastava et al. (2013).

Similarly, under abiotic stress conditions, expression of stress-related genes was elevated in both *CaBiP1*-overexpressing and WT *Arabidopsis* lines, but levels were higher in the former (**Figures 7**–**10**). Transcription of DREB2A (a major transcription factor functioning under stress conditions, Nakashima et al., 2000) and APX2 (an important H_2_O_2_-scavenging enzyme, Suzuki et al., 2013) increased continuously throughout the treatment of salt, osmotic and drought stress (**Figures 8**–**10**), which suggests overexpression of *CaBiP1* promote plant responsivity to water deficit and the ability to remove ROS. Expression level of HsfA2, a regulatory amplifier of heat-response genes (Schramm et al., 2006), peaked at 3 h then fell down under the treatment of heat and osmotic stress (**Figures 7**–**9**), which implies that overexpression of *CaBiP1* enhances the function of HsfA2 by up-regulating its expression level but not changing its expression pattern. Expression of RD29A, a marker for plant response to water deficit stress (Msanne et al., 2011), kept increasing throughout the treatment of salt and drought stress, while peaked at 3 h then fell down under osmotic stress (**Figures 8**–**10**), which hints that RD29A is more responsive to salt and drought stress, and overexpression of *CaBiP1* advancing this responsivity of RD29A. Based on the discussion above, it can be assumed CaBiP1 improves plant tolerance to environmental stresses by the up-regulating stress-responsive genes, but elucidating the exact mechanisms and signaling pathways involved requires need further study.

## Conclusion

BiP plays important roles in helping plants to cope with ER stress induced by adverse environmental conditions. In this study, we characterized the sequence and expression patterns of three *BiP* genes in pepper. While *CaBiP1* and *CaBiP2* were constitutively expressed in all tissues under normal and stressed conditions, *CaBiP3* was mainly transcribed under stress treatments. The silencing of *CaBiP1* lowered the tolerance of pepper plants to ER and environmental stresses, whereas overexpression of *CaBiP1* in *Arabidopsis* enhanced of tolerance to these stresses. Our study suggests that CaBiP1 may contribute to tolerance to abiotic stress in pepper by reducing ROS accumulation, increasing the water retention ability, and enhancing the UPR pathways and expression of stress-related genes.

## Author Contributions

HW and ML designed the experiments. HW, HN, and YZ performed the research. HW drafted the manuscript. LM revised the paper and contributed reagents/materials/analysis tools. All authors read and approved the final manuscript.

## Conflict of Interest Statement

The authors declare that the research was conducted in the absence of any commercial or financial relationships that could be construed as a potential conflict of interest.

## Footnotes

1 http://peppersequence.genomics.cn/page/species/index.jsp

2 http://peppergenome.snu.ac.kr

3 http://www.ncbi.nlm.nih.gov/

4 http://rice.plantbiology.msu.edu/

5 http://www.ebi.ac.uk/Tools/msa/clustalo/

6 http://meme-suite.org/tools/meme

7 http://gsds.cbi.pku.edu.cn

8 https://www.ncbi.nlm.nih.gov/tools/primer-blast/

## Supplementary Material

FIGURE S1 Confirmation of *CaBiP1*-silenced pepper seedlings and *CaBiP1*-overexpressing *Arabidopsis* lines. **(A)** Phenotypes of *CaBiP1*-silenced pepper seedlings. *CaPDS*, phytoene desaturase gene for chlorophyll synthesis; TRV2:00, control plants harboring the empty TRV2 vector. **(B)** Measurement of the silencing efficiency for *CaBiP1* expression in silenced pepper seedlings at the 30th day after inoculation. **(C)** Screening of *CaBiP1*-overexpressing *Arabidopsis* lines in MS medium containing kanamycin. **(D)** Measurement of *CaBiP1* expression levels in *CaBiP1*-overexpressing *Arabidopsis* lines by semi-quantitative PCR. The *AtActin2* gene was used as the internal control. Error bars represent standard deviation from three biological replicates.

FIGURE S2 Enhanced tolerance to ER stress induced by DTT in *CaBiP1*-overexpressing *Arabidsopsis* lines. **(A)** Germination rate on MS medium with 3 mM DTT. **(B)** Fresh weight of 5-day-old *CaBiP1*-OE *Arabidsopsis* seedlings grown on MS medium with 2 mM DTT for 15 days. Data represent the mean ± standard deviation from three biological replicates. Statistical significance is indicated as by a single asterisk (*p* < 0.05) and double asterisk (*p* < 0.01) based on the results of the Student’s *t*-test.

FIGURE S3 Seed germination rate in WT and *CaBiP1*-OE (OE7 and OE8) *Arabidsopsis* lines grown on MS medium with 100 mM NaCl. Data represent the mean ± standard deviation from three biological replicates. Statistical significance is indicated by a single asterisk (*p* < 0.05) based on the results of the Student’s *t*-test.

## Abbreviations

BiP: binding proteins
DAB: diaminobenzidine
ER: endoplasmic reticulum
GFP: green fluorescent protein
Hsp: heat shock proteins
MDA: malondialdehyde
ORF: open reading frame
qRT-PCR: quantitative real-time PCR
REL: relative electric leakage
RWC: relative water content
UPR: unfolding protein response.

## References

[B1] Alvim F. C. Carolino S. M. B. Cascardo J. C. M. Nunes C. C. Martinez C. A. Otoni W. C. (2001). Enhanced accumulation of BiP in transgenic plants confers tolerance to water stress. *Plant Physiol.* 126 1042–1054. 10.1104/pp.126.3.1042 11457955 PMC116461

[B2] Bertolotti A. Zhang Y. Hendershot L. M. Harding H. P. Ron D. (2000). Dynamic interaction of BiP and ER stress transducers in the unfolded-protein response. *Nat. Cell. Biol.* 2 326–332. 10.1038/35014014 10854322

[B3] Bradford M. M. (1976). A rapid and sensitive method for the quantitation of microgram quantities of protein utilizing the principle of protein-dye binding. *Anal. Biochem.* 72 248–254. 10.1016/0003-2697(76)90527-3 942051

[B4] Carvalho H. H. Brustolini O. J. B. Pimenta M. R. Mendes G. C. Gouveia B. C. Silva P. A. (2014a). The molecular chaperone binding protein BiP prevents leaf dehydration-induced cellular homeostasis disruption. *PLoS ONE* 9:e86661 10.1371/journal.pone.0086661 PMC3906070 24489761

[B5] Carvalho H. H. Silva P. A. Mendes G. C. Brustolini O. J. B. Pimenta M. R. Gouveia B. C. (2014b). The endoplasmic reticulum binding protein BiP displays dual function in modulating cell death events. *Plant Physiol.* 164 654–670. 10.1104/pp.113.231928 24319082 PMC3912096

[B6] Chakraborty K. Sairam R. K. Bhattacharya R. C. (2012). Differential expression of salt overly sensitive pathway genes determines salinity stress tolerance in *Brassica* genotypes. *Plant Physiol. Biochem.* 51 90–101. 10.1016/j.plaphy.2011.10.001 22153244

[B7] Cheng Q. Zhou Y. Liu Z. Zhang L. Song G. Guo Z. (2015). An alternatively spliced heat shock transcription factor, *OsHSFA2dl*, functions in the heat stress-induced unfolded protein response in rice. *Plant Biol.* 17 419–429. 10.1111/plb.12267 25255693

[B8] Clough S. J. Bent A. F. (1998). Floral dip: a simplified method for *Agrobacterium*-mediated transformation of *Arabidopsis thaliana*. *Plant J.* 16 735–743. 10.1046/j.1365-313x.1998.00343.x 10069079

[B9] Dang F. F. Wang Y. N. Yu L. Eulgem T. Lai Y. Liu Z. Q. (2013). CaWRKY40, a WRKY protein of pepper, plays an important role in the regulation of tolerance to heat stress and resistance to *Ralstonia solanacearum* infection. *Plant Cell Environ.* 36 757–774. 10.1111/pce.12011 22994555

[B10] Denecke J. Goldman M. H. S. Demolder J. Seurinck J. Botterman J. (1991). The tobacco luminal binding protein is encoded by a multigene family. *Plant Cell* 3 1025–1035. 10.1105/tpc.3.9.1025 1822990 PMC160068

[B11] Deng Y. Humbert S. Liu J. X. Srivastava R. Rothstein S. J. Howell S. H. (2011). Heat induces the splicing by IRE1 of a mRNA encoding a transcription factor involved in the unfolded protein response in *Arabidopsis*. *Proc. Natl. Acad. Sci. U.S.A.* 108 7247–7252. 10.1073/pnas.1102117108 21482766 PMC3084119

[B12] Deng Y. Srivastava R. Howell S. H. (2013). Endoplasmic reticulum (ER) stress response and its physiological roles in plants. *Int. J. Mol. Sci.* 14 8188–8212. 10.3390/ijms14048188 23591838 PMC3645738

[B13] Dhindsa R. S. Thorpe T. A. (1981). Leaf Senescence: Correlated with increased levels of membrane permeability and lipid peroxidation, and decreased levels of superoxide dismutase and catalase. *J. Exp. Bot.* 32 93–101. 10.1093/jxb/32.1.93

[B14] Fanata D. W. I. Lee S. Y. Lee K. O. (2013). The unfolded protein response in plants: a fundamental adaptive cellular response to internal and external stresses. *J. Proteomics* 93 356–368. 10.1016/j.jprot.2013.04.023 23624343

[B15] Guo M. Liu J. H. Ma X. Zhai Y. F. Gong Z. H. Lu M. H. (2016). Genome-wide analysis of the *Hsp70* family genes in pepper (*Capsicum annuum* L.) and functional identification of *CaHsp70-2* involvement in heat stress. *Plant Sci.* 252 246–256. 10.1016/j.plantsci 27717461

[B16] Guo M. Zhai Y. F. Lu J. P. Chai L. Chai W. G. Gong Z. H. (2014). Characterization of *CaHsp70-1*, a pepper heat-shock protein gene in response to heat stress and some regulation exogenous substances in *Capsicum annuum* L. *Int. J. Mol. Sci.* 15 19741–19759. 10.3390/ijms151119741 25356507 PMC4264136

[B17] Howell S. H. (2013). Endoplasmic reticulum stress responses in plants. *Annu. Rev. Plant. Biol.* 64 477–499. 10.1146/annurev-arplant-050312-120053 23330794

[B18] Jung K. H. Gho H. J. Nguyen M. X. Kim S. R. An G. (2013). Genome-wide expression analysis of *HSP70* family genes in rice and identification of a cytosolic *HSP70* gene highly induced under heat stress. *Funct. Integr. Gen.* 13 391–402. 10.1007/s10142-013-0331-6 23852542

[B19] Kim S. Park M. Yeom S. I. Kim Y. M. Lee J. M. Lee H. A. (2014). Genome sequence of the hot pepper provides insights into the evolution of pungency in *Capsicum* species. *Nat. Genet* 46 270–278. 10.1038/ng.2877 24441736

[B20] Leborgne-Castel N. Jelitto-Van Dooren E. P. Crofts A. J. Denecke J. (1999). Overexpression of BiP in tobacco alleviates endoplasmic reticulum stress. *Plant Cell* 11 459–470. 10.1105/tpc.11.3.459 10072404 PMC144191

[B21] Li Y. Humbert S. Howell S. H. (2012). ZmbZIP60 mRNA is spliced in maize in response to ER stress. *BMC Res. Notes* 5:144 10.1186/1756-0500-5-144 PMC3369818 22417282

[B22] Liebrand T. W. Kombrink A. Zhang Z. Sklenar J. Jones A. M. Robatzek S. (2014). Chaperones of the endoplasmic reticulum are required for ve1-mediated resistance to verticillium. *Mol. Plant Pathol.* 15 109–117. 10.1111/mpp.12071 24015989 PMC6638731

[B23] Liu J. X. Howell S. H. (2010). bZIP28 and NF-Y transcription factors are activated by ER stress and assemble into a transcriptional complex to regulate stress response genes in *Arabidopsis*. *Plant Cell* 22 782–796. 10.1105/tpc.109.072173 20207753 PMC2861475

[B24] Livak K. J. Schmittgen T. D. (2001). Analysis of relative gene expression data using real-time quantitative PCR and the 2-ΔΔCT Method. *Methods* 25 402–408. 10.1006/meth.2001.1262 11846609

[B25] Maruyama D. Endo T. Nishikawa S. (2015). BiP3 supports the early stages of female gametogenesis in the absence of BiP1 and BiP2 in *Arabidopsis thaliana*. *Plant. Signal. Behav.* 10:e1035853 10.1080/15592324.2015.1035853 PMC4622982 26251880

[B26] Maruyama D. Sugiyama T. Endo T. Nishikawa S. (2014). Multiple BiP genes of *Arabidopsis thaliana* are required for male gametogenesis and pollen competitiveness. *Plant Cell Physiol.* 55 801–810. 10.1093/pcp/pcu018 24486762

[B27] Msanne J. Lin J. Stone J. M. Awada T. (2011). Characterization of abiotic stress-responsive *Arabidopsis thaliana* RD29A and RD29B genes and evaluation of transgenes. *Planta* 234 97–107. 10.1007/s00425-011-1387-y 21374086

[B28] Nakashima K. Shinwari Z. K. Sakuma Y. Seki M. Miura S. Shinozaki K. (2000). Organization and expression of two Arabidopsis DREB2 genes encoding DRE-binding proteins involved in dehydration- and high-salinity-responsive gene expression. *Plant Mol. Biol.* 42 657–665. 10.1023/A:1006321900483 10809011

[B29] Noh S. J. Kwon C. S. Oh D. H. Moon J. S. Chung W. I. (2003). Expression of an evolutionarily distinct novel BiP gene during the unfolded protein response in *Arabidopsis thaliana*. *Gene* 311 81–91. 10.1016/S0378-1119(03)00559-6 12853141

[B30] Ozgur R. Turkan I. Uzilday B. Sekmen A. H. (2014). Endoplasmic reticulum stress triggers ROS signalling, changes the redox state, and regulates the antioxidant defence of *Arabidopsis thaliana*. *J. Exp. Bot.* 65 1377–1390. 10.1093/jxb/eru034 24558072 PMC3969530

[B31] Qin C. Yu C. S. Shen Y. O. Fang X. D. Chen L. Min J. M. (2014). Whole-genome sequencing of cultivated and wild peppers provides insights into *Capsicum* domestication and specialization. *Proc. Natl. Acad. Sci. U.S.A* 111 5135–5140. 10.1073/pnas.1400975111 24591624 PMC3986200

[B32] Sarkar N. K. Kundnani P. Grover A. (2013). Functional analysis of Hsp70 superfamily proteins of rice (*Oryza sativa*). *Cell Stress Chaperones* 18 427–437. 10.1007/s12192-012-0395-6 23264228 PMC3682022

[B33] Schramm F. Ganguli A. Kiehlmann E. Englich G. Walch D. von Koskull-Döring P. (2006). The heat stress transcription factor HsfA2 serves as a regulatory amplifier of a subset of genes in the heat stress response in *Arabidopsis*. *Plant Mol. Biol.* 60 759–772. 10.1007/s11103-005-5750-x 16649111

[B34] Shen J. Chen X. Hendershot L. Prywes R. (2002). ER stress regulation of ATF6 localization by dissociation of BiP/GRP78 binding and unmasking of Golgi localization signals. *Dev. Cell* 3 99–111. 10.1016/S1534-5807(02)00203-4 12110171

[B35] Srivastava R. Deng Y. Shah S. Rao A. G. Howell S. H. (2013). Binding protein is a master regulator of the endoplasmic reticulum stress sensor/transducer bZIP28 in *Arabidopsis*. *Plant Cell* 25 1416–1429. 10.1105/tpc.113.110684 23624714 PMC3663277

[B36] Suzuki N. Miller G. Sejima H. Harper J. Mittler R. (2013). Enhanced seed production under prolonged heat stress conditions in *Arabidopsis thaliana* plants deficient in cytosolic ascorbate peroxidase 2. *J. Exp. Bot.* 64 253–263. 10.1093/jxb/ers335 23183257 PMC3528037

[B37] Tamura K. Stecher G. Peterson D. Filipski A. Kumar S. (2013). MEGA6: molecular evolutionary genetics analysis version 6.0. *Mol. Biol. Evol.* 30 2725–2729. 10.1093/molbev/mst197 24132122 PMC3840312

[B38] Thiry A. A. Dulanto P. N. C. Reynolds M. P. Davies W. J. (2016). How can we improve crop genotypes to increase stress resilience and productivity in a future climate? a new crop screening method based on productivity and resistance to abiotic stress. *J. Exp.Bot.* 67 5593–5603. 10.1093/jxb/erw330 27677299 PMC5066489

[B39] Valente M. A. S. Faria J. A. Q. A. Soares-Ramos J. R. L. Reis P. A. B. Pinheiro G. L. Piovesan N. D. (2009). The ER luminal binding protein (BiP) mediates an increase in drought tolerance in soybean and delays drought-induced leaf senescence in soybean and tobacco. *J. Exp. Bot.* 60 533–546. 10.1093/jxb/ern296 19052255 PMC2651463

[B40] Wakasa Y. Hayashi S. Takaiwa F. (2012). Expression of *OsBiP4* and *OsBiP5* is highly correlated with the endoplasmic reticulum stress response in rice. *Planta* 236 1519–1527. 10.1007/s00425-012-1714-y 22824965

[B41] Wan S. Jiang L. (2016). Endoplasmic reticulum (ER) stress and the unfolded protein response (UPR) in plants. *Protoplasma* 253 753–764. 10.1007/s00709-015-0842-1 26060134

[B42] Wang J. E. Liu K. K. Li D. W. Zhang Y. L. Zhao Q. He Y. M. (2013). A novel peroxidase *CanPOD* gene of pepper is involved in defense responses to *Phytophthora capsici* infection as well as abiotic stress tolerance. *Int. J. Mol. Sci.* 14 3158–3177. 10.3390/ijms14023158 23380961 PMC3588037

[B43] Yang X. Srivastava R. Howell S. H. Bassham D. C. (2016). Activation of autophagy by unfolded proteins during endoplasmic reticulum stress. *Plant J.* 85 83–95. 10.1111/tpj.13091 26616142

[B44] Yin Y. X. Guo W. L. Zhang Y. L. Ji J. J. Xiao H. J. Yan F. (2014). Cloning and characterisation of a pepper aquaporin, CaAQP, which reduces chilling stress in transgenic tobacco plants. *Plant Cell Tissue Organ. Cult.* 118 431–444. 10.1007/s11240-014-0495-3

[B45] Zhang L. Zhao H. K. Dong Q. L. Zhang Y. Y. Wang Y. M. Li H. Y. (2015). Genome-wide analysis and expression profiling under heat and drought treatments of *HSP70* gene family in soybean (*Glycine max* L.). *Front. Plant Sci.* 6:773 10.3339/fpls.2015.00773 PMC4585176 26442082

[B46] Zhu J. Hao P. Chen G. Han C. Li X. Zeller F. J. (2014). Molecular cloning, phylogenetic analysis, and expression profiling of endoplasmic reticulum molecular chaperone *BiP* genes from bread wheat (*Triticum aestivum* L.). *BMC Plant Biol.* 14:260 10.1186/s12870-014-0260-0 PMC4189733 25273817

