# Supplementary figures and images for: Characterization of BiP Genes from Pepper (Capsicum annuum L.) and the Role of CaBiP1 in Response to Endoplasmic Reticulum and Multiple Abiotic Stresses
Source: Front Plant Sci. 2017 Jun 28;8:1122. doi: 10.3389/fpls.2017.01122 (PMC5487487; doi:10.3389/fpls.2017.01122)

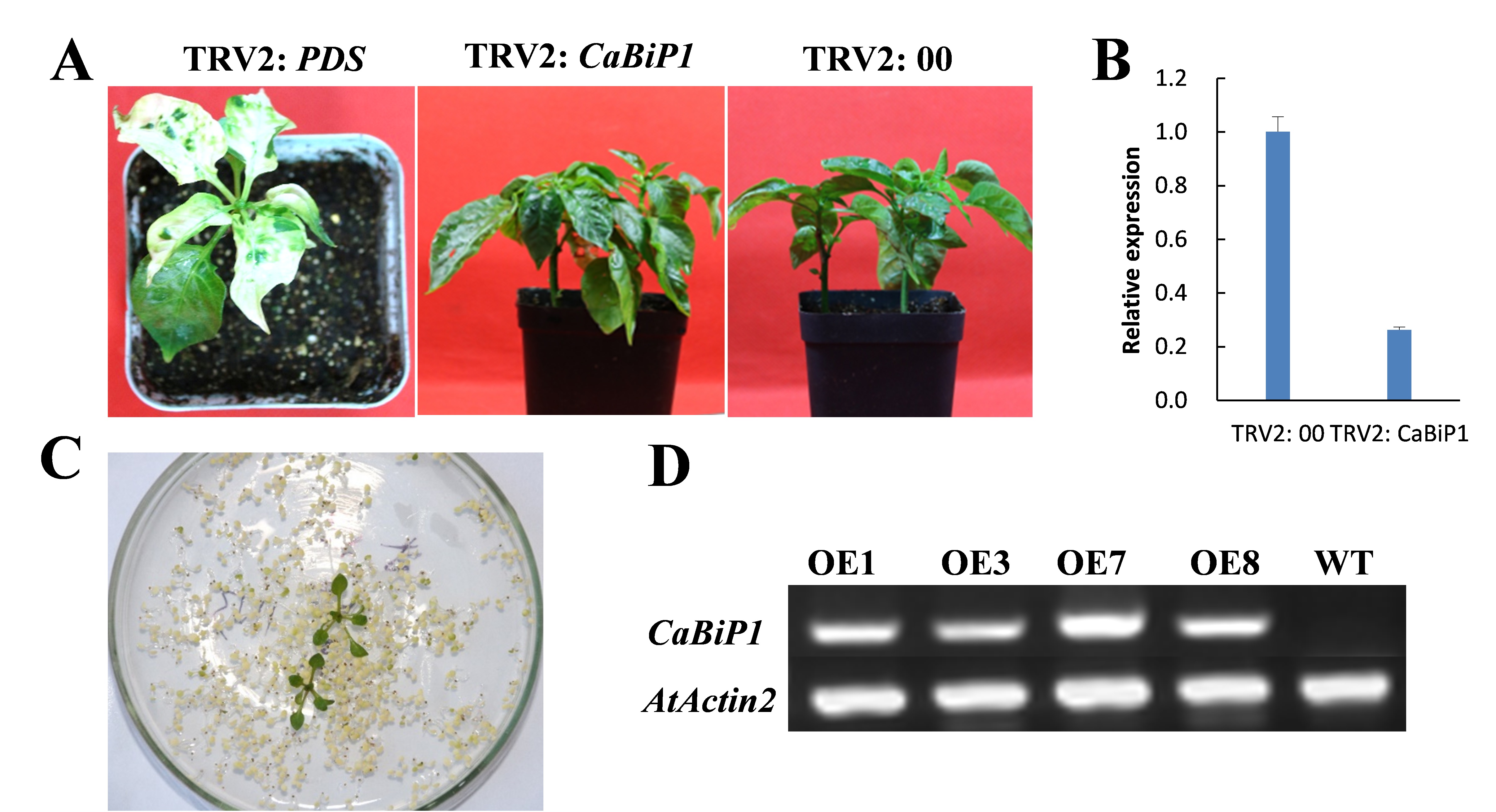

Supplement: FIGURE S1 — Confirmation of CaBiP1-silenced pepper seedlings and CaBiP1-overexpressing Arabidopsis lines. (A) Phenotypes of CaBiP1-silenced pepper seedlings. CaPDS, phytoene desaturase gene for chlorophyll synthesis; TRV2:00, control plants harboring the empty TRV2 vector. (B) Measurement of the silencing efficiency for CaBiP1 expression in silenced pepper seedlings at the 30th day after inoculation. (C) Screening of CaBiP1-overexpressing Arabidopsis lines in MS medium containing kanamycin. (D) Measurement of CaBiP1 expression levels in CaBiP1-overexpressing Arabidopsis lines by semi-quantitative PCR. The AtActin2 gene was used as the internal control. Error bars represent standard deviation from three biological replicates. [file Image_1.JPEG]

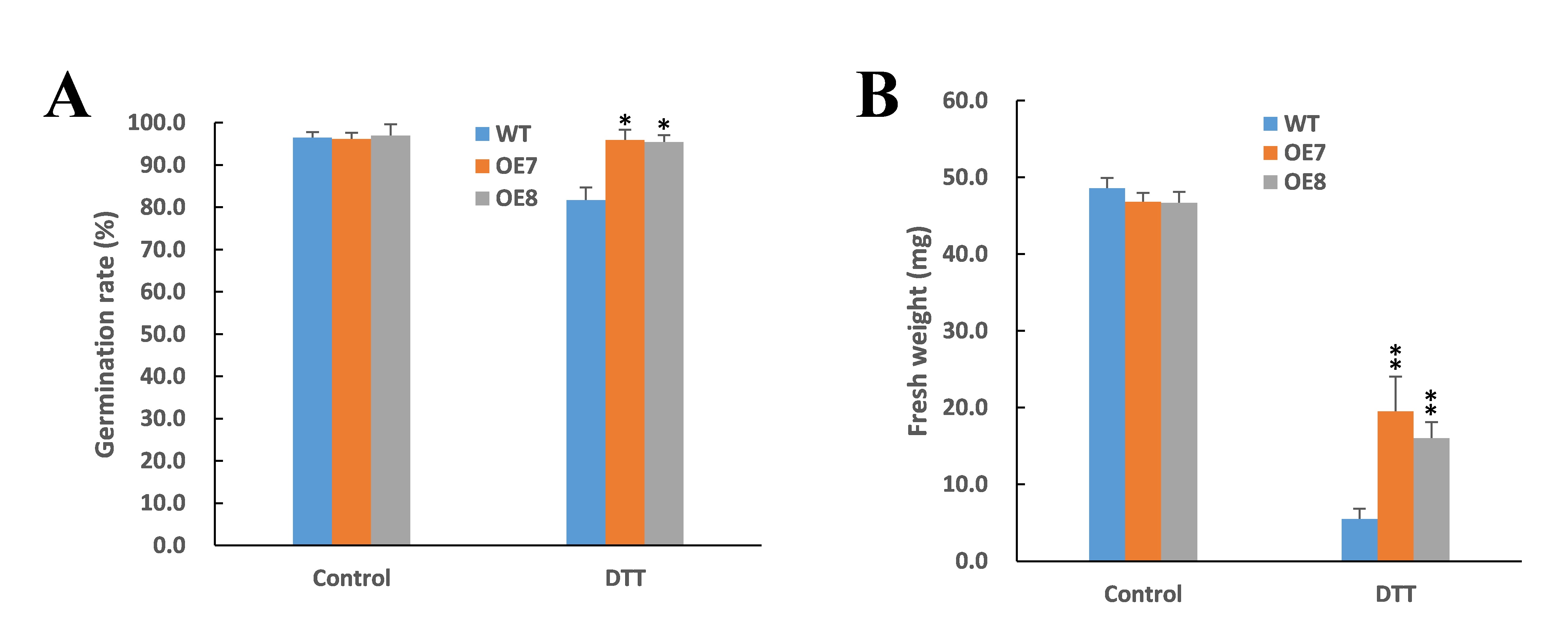

Supplement: FIGURE S2 — Enhanced tolerance to ER stress induced by DTT in CaBiP1-overexpressing Arabidsopsis lines. (A) Germination rate on MS medium with 3 mM DTT. (B) Fresh weight of 5-day-old CaBiP1-OE Arabidsopsis seedlings grown on MS medium with 2 mM DTT for 15 days. Data represent the mean ± standard deviation from three biological replicates. Statistical significance is indicated as by a single asterisk (p < 0.05) and double asterisk (p < 0.01) based on the results of the Student’s t-test. [file Image_2.JPEG]

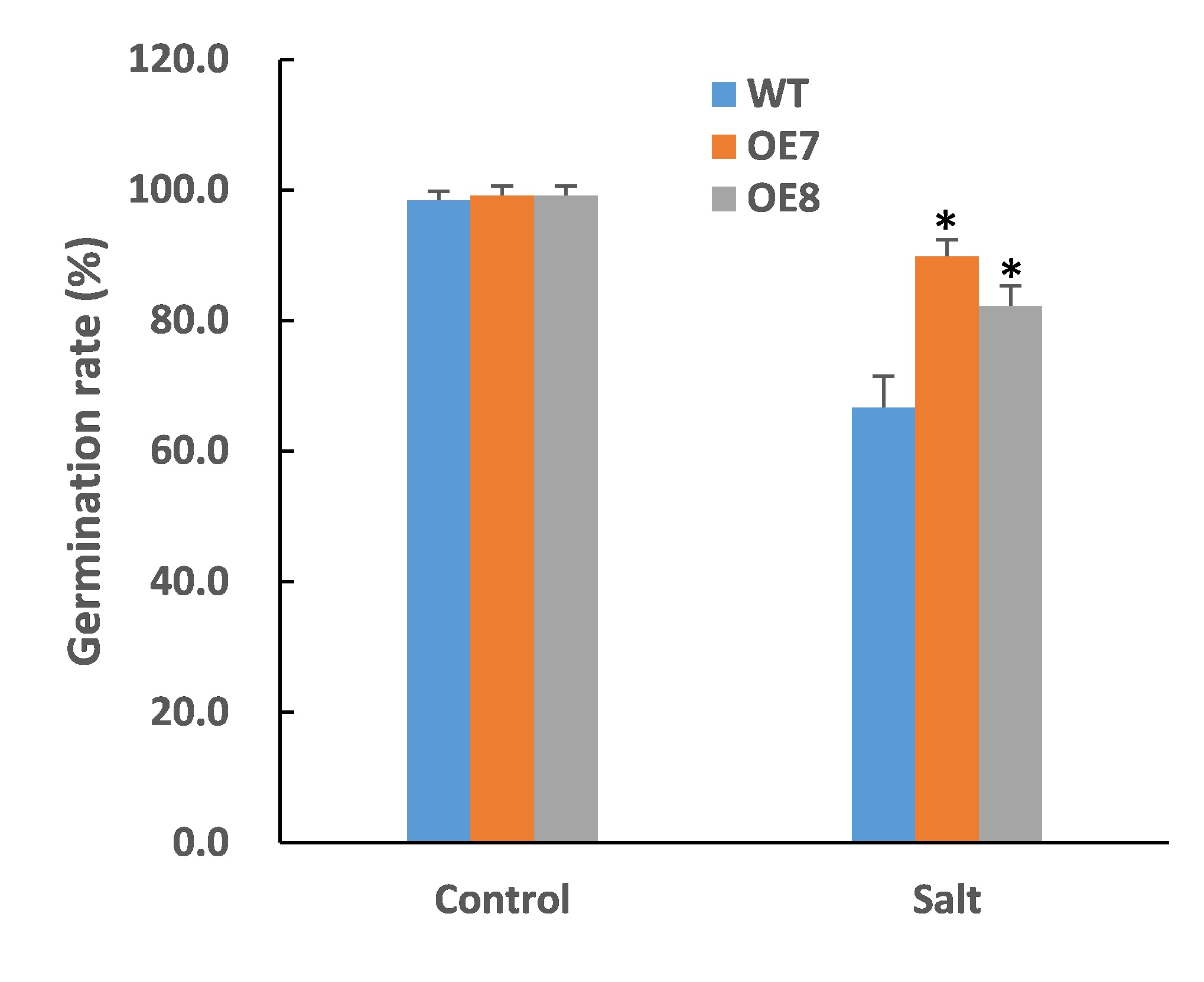

Supplement: FIGURE S3 — Seed germination rate in WT and CaBiP1-OE (OE7 and OE8) Arabidsopsis lines grown on MS medium with 100 mM NaCl. Data represent the mean ± standard deviation from three biological replicates. Statistical significance is indicated by a single asterisk (p < 0.05) based on the results of the Student’s t-test. [file Image_3.JPEG]
