# Supplementary material for: Characterization of BiP Genes from Pepper (Capsicum annuum L.) and the Role of CaBiP1 in Response to Endoplasmic Reticulum and Multiple Abiotic Stresses
Source: Front Plant Sci. 2017 Jun 28;8:1122. doi: 10.3389/fpls.2017.01122 (PMC5487487; doi:10.3389/fpls.2017.01122)
Supplement: Supplementary file 4 [file Table_1.DOCX]

**Table S1.** Sequence of primers

| **Primers** | **Sequence(5'–3')** |
| --- | --- |
| cDNA-CaBiP1-F | ATGGCTGGTCATTGTAGGC |
| cDNA-CaBiP1-R | CAGCTCATCATGGCCATCT |
| qCaUBI3-F | TGTCCATCTGCTCTCTGTTG |
| qCaUBI3-R | CACCCCAAGCACAATAAGAC |
| qCaBiP1-F | AGAGATCCCTCAGTAGCCAGC |
| qCaBiP1-R | GTTGTTCAACTCCTCAAAACGT |
| qCaBiP2-F | AAGAAGTTGAGGCAGTGTGC |
| qCaBiP2-R | TGTGAATCGTCATCATCGTTG |
| qCaBiP3-F | CAACATACTCTTGTGTGGGCG |
| qCaBiP3-R | TGAAGGGGTGATTCTGTTTCC |
| TRV2-CaBiP1-F | TGAAGATGGCCATGATGAGC |
| TRV2-CaBiP1-R | GTAGATTCCAAGTATTGGCACTTG |
| GFP-CaBiP1-F | ATGGCTGGTCATTGTAGGC |
| GFP-CaBiP1-R | CAGCTCATCATGGCCATCT |
| qAtHsp70-F | TGCTGGAGGTGTTATGACCA |
| qAtHsp70-R | GACTCCTCTTGGTGCTGGAG |
| qAtHsp101-F | CAGGGCTAACTGGGAAAGTAACA |
| qAtHsp101-R | CACCTCCCGCATCACACA |
| qAtAPX-F | AATATGCTGCAGATGAGGATGC |
| qAtAPX-R | CAAGAATCAAGGAGGTAGGAGATG |
| qAtActin2-F | CTTCGTCTTCCACTTCAG |
| qAtActin2-R | ATCATACCAGTCTCAACAC |
| qAtRD29A-F | ACTCAACACACACCAGCAGCACC |
| qAtRD29A-R | GCTCATGCTCATTGCTTTGTCCA |
| qAtbZIP28-F | CCGCCGCTAGATCCTCTTTT |
| qAtbZIP28-R | GCCGCTTCTGTTTTCCGATG |
| qAtbZIP60F-F | GAAGGAGACGATGATGCTGTGGCT |
| qAtbZIP60F-R | GCAGGGATTCCAACAAGAGCACAG |
| qAtbZIP60S-F | GAAGGAGACGATGATGCTGTGGCT |
| qAtbZIP60S-R | AGCAGGGAACCCAACAGCAGACT |
| qAtNFY-C2-F | CAGTCAGAAGAGGGTCAACA |
| qAtNFY-C2-R | AAATCAGTGGTATGCTCGA |
| qAtDREB2A-F | AAGGATTTGGGGTAAATGGGTTG |
| qAtDREB2A-R | CAGCCTCATCATAAGCAGAAGCA |
| qAtHsfA2-F | GGTTCTGTAGCGGCTTCTTCAT |
| qAtHsfA2-R | TGGTGGCCCTGTTTCGTTA |
| qAtHsfA7a-F | GCAAACGAAGAGTTTTTGTT |
| qAtHsfA7a-R | ATCTCTTTGTTTCAACAACTGAT |
