# Supplementary material for: Characterization of BiP Genes from Pepper (Capsicum annuum L.) and the Role of CaBiP1 in Response to Endoplasmic Reticulum and Multiple Abiotic Stresses
Source: Front Plant Sci. 2017 Jun 28;8:1122. doi: 10.3389/fpls.2017.01122 (PMC5487487; doi:10.3389/fpls.2017.01122)
Supplement: Supplementary file 5 [file Table_2.DOCX]

**Table S2.** The amino acid sequences of conserved motifs

| **Motif** | **Width（aa）** | **Best Possible Match** |
| --- | --- | --- |
| 1 | 200 | FSPEEISAMILTKMKETAEAYLGKTIKDAVVTVPAYFNDAQRQATKDAGIIAGLNVARIINEPTAAAIAYGLDKKGGEKNILVFDLGGGTFDVSILTIDNGVFEVLATNGDTHLGGEDFDQRIMEYFIKLIKKKHGKDISKDNKALGKLRRECERAKRALSSQHQVRVEIESLFDGVDFSEPLTRARFEELNNDLFRKTM |
| 2 | 200 | QIDEIVLVGGSTRIPKVQQLLKDYFDGKEPNKGVNPDEAVAYGAAVQGGILSGEGGDETKDILLLDVAPLTLGIETVGGVMTKLIPRNTVIPTKKSQVFTTYQDQQTTVTIQVYEGERSLTKDCRNLGKFDLTGIPPAPRGTPQIEVTFEVDANGILNVKAEDKASGKSEKITITNDKGRLSQEEIERMVKEAEEFAEED |
| 3 | 80 | HVEIIANDQGNRITPSWVAFTDSERLIGEAAKNQAAVNPERTIFDVKRLIGRKFDDKEVQRDMKLVPYKIVNKDGKPYIQ |
| 4 | 54 | DEKEKIETATKEALEWLDDNQSAEKEDYDEKLKEVEAVCNPIITAVYQRSGGAP |
| 5 | 21 | LGTVIGIDLGTTYSCVGVYKN |
| 6 | 26 | KVKERVDARNSLETYVYNMKNQINDK |
| 7 | 28 | MAGHWRRRNSLFVFAFVLFGCFFAFSIA |
| 8 | 9 | PVKKAMEDA |
| 9 | 4 | HDEL |

Motif numbers corresponded to the motifs in Figure 2B.
